# Leukocyte-Poor Platelet-Rich Plasma Injections Improve Cartilage T1ρ and T2 and Patient-Reported Outcomes in Mild-to-Moderate Knee Osteoarthritis

**DOI:** 10.1016/j.asmr.2023.04.009

**Published:** 2023-05-20

**Authors:** Favian Su, Michelle W. Tong, Drew A. Lansdown, Anthony Luke, C. Benjamin Ma, Brian T. Feeley, Sharmila Majumdar, Alan L. Zhang

**Affiliations:** a Department of Orthopaedic Surgery, University of California San Francisco, San Francisco, California, U.S.A.; b Musculoskeletal Quantitative Imaging Research, Department of Radiology and Biomedical Imaging, University of California San Francisco, San Francisco, California, U.S.A.

## Abstract

**Purpose:**

To use T1ρ and T2 magnetic resonance imaging to evaluate the effect of leukocyte-poor platelet-rich plasma (LP-PRP) injections on knee cartilage health and to correlate structural changes with patient-reported outcome measurements.

**Methods:**

Ten patients with symptomatic unilateral mild-to-moderate knee osteoarthritis (Kellgren-Lawrence Grade 1-2) underwent T1ρ and T2 magnetic resonance imaging of both the symptomatic and contralateral knee before injection and 6 months after injection with LP-PRP. Patient-reported outcome questionnaires (Knee Osteoarthritis Outcome Score and International Knee Documentation Committee) that evaluate the domains of pain, symptoms, activities of daily living, sports function, and quality of life were completed at baseline, 3 months, 6 months, and 12 months after injection. T1ρ and T2 relaxation times, which are correlated with the proteoglycan and collagen concentration of cartilage, were measured in compartments with and without chondral lesions.

**Results:**

Ten patients were prospectively enrolled (9 female, 1 male) with a mean age of 52.9 years (range, 42-68) years and mean body mass index of 23.2 ± 1.9. Significant increases in Knee Osteoarthritis Outcome Score for all subscales and International Knee Documentation Committee scores were observed 3 months after injection and the improvements were sustained at 12 months. T1ρ and T2 values of compartments with chondral lesions were observed to significantly decrease by 6.0% (*P* = .036) and 7.1% (*P* = .017) 6 months after LP-PRP injection, respectively. No significant associations between T1ρ and T2 relaxation times and improvement in patient-reported outcomes were observed.

**Conclusions:**

Patients undergoing LP-PRP injections for the treatment of mild-to-moderate knee osteoarthritis had increased proteoglycan and collagen deposition in the cartilage of affected compartments by 6 months after injection. Patient-reported outcomes scores improved 3 months after injection and were sustained through 1 year after injection, but these improvements were not associated with the changes in proteoglycan and collagen deposition in knee cartilage.

**Level of Evidence:**

Level II, prospective cohort study.

Knee osteoarthritis (OA) is a painful and debilitating condition estimated to affect approximately 21 million patients in the United States.^1^^,^^2^ Despite this widespread prevalence that is growing rapidly due to the aging population, there are no current preventative treatment options or established disease-modifying therapies.^3^^,^^4^ There has been recent interest in the use of biologic therapies such as platelet-rich plasma (PRP) injections to treat OA. Specifically, leukocyte-poor platelet-rich plasma (LP-PRP) concentrate is rich in anabolic cytokines that may promote cartilage healing while removing proinflammatory catabolic cytokines.^5^, ^6^, ^7^ The use of LP-PRP injections in the treatment of early knee OA has demonstrated good clinical outcomes in multiple Level I trials compared with placebo and hyaluronic acid injections.^8^^,^^9^ It is unknown, however, whether the changes to patient symptoms from LP-PRP treatment are due to analgesic effects or whether clinical improvements are due to biologic alteration of the natural course of cartilage degeneration and prevention of OA progression.

Standard magnetic resonance imaging (MRI) techniques are useful in detecting morphologic changes associated with cartilage breakdown but are insensitive in detecting initial changes in the cartilage matrix.^10^^,^^11^ Over the past decade, T1ρ and T2 quantitative magnetic resonance imaging (QMRI) sequences have been shown to be reliable in identifying early changes in the proteoglycan and collagen composition of cartilage, respectively.^12^^,^^13^ Elevated T1ρ and T2 relaxation times have been correlated with a decrease in the proteoglycan and collagen concentration of cartilage in osteoarthritic knees.^14^ To date, several studies have evaluated the cartilage collagen matrix after PRP injection using T2 mapping, but the results have been mixed.^15^, ^16^, ^17^ Moreover, the effects of PRP treatment on the T1ρ of knee cartilage in the setting of early knee OA is also unclear. Assessment of whether PRP can modify or prevent cartilage degeneration may potentially allow for substantiation of this novel treatment for knee OA.

The purposes of this study were to use T1ρ and T2 MRI to evaluate the effect of LP-PRP injections on knee cartilage health and to correlate structural changes with patient reported outcomes measurements. We hypothesized that LP-PRP injections would improve patient-reported outcomes and T1ρ and T2 relaxation times after treatment compared with before treatment and that the improvement in patient-reported outcomes would be correlated with the improvement in T1ρ and T2 relaxation times.

## Methods

### Study Participants

This prospective cohort study was conducted after obtaining approval from our institutional review board. Ten subjects with unilateral symptomatic knee OA provided full informed consent and were enrolled. Patients were included if they were between the ages of 18 and 70 years, had radiographic evidence of mild-to-moderate knee OA (Kellgren-Lawrence [KL] grade 1-2) in any compartment (medial, lateral, and/or patellofemoral), had a body mass index less than 30, and had not responded to conservative treatment with physical therapy for at least 6 weeks. Exclusion criteria included patients with end-stage OA (KL grade 3-4) in any compartment, symptomatic meniscus tears (flap, bucket handle, root tear), inflammatory arthropathy, previous surgery on the affected knee, or previous knee injections with corticosteroid, hyaluronic acid, PRP, or stem cells.

MRI scans were performed preinjection and at 6 months’ postinjection for the symptomatic knee and the contralateral knee (to be used as a control) simultaneously. At 6-month follow-up, 8 patients had completed MRI scans, and 7 had both MRI and patient-reported outcome data. At 12-month follow-up, 1 subject had elected to undergo arthroscopic knee surgery, leaving 7 patients with complete MRI and patient-reported outcome data.

### LP-PRP Injection Protocol

An LP-PRP injection was prepared using Regenkit BCT (RegenLab, New York, NY). First, 9 mL of venous blood was drawn using a 21-G butterfly needle from the antecubital vein into a tube containing thixotropic gel for blood component separation. The tube was then centrifuged at 1500 relative centrifuge force for 9 minutes to isolate the PRP from the red and white blood cells. Compared with baseline values in peripheral blood, the product monograph reported that approximately 80% of platelets were recovered, whereas 99.7% of red blood cells and 87% to 90% of white blood cells were depleted.^18^ The cellular elements were concentrated by a factor of 1.6, 0.007, and 0.2 for platelets, red blood cells, and white blood cells, respectively.^18^ Whole blood cell counts obtained within 3 months before study enrollment were available for 6 patients. The mean platelet, leukocyte, and red blood cell counts were 241 × 10^3^/μL (range, 148 × 10^3^/μL to 300 × 10^3^/μL), 5.35 × 10^3^/μL (range, 4.2 × 10^3^/μL to 6.5 × 10^3^/μL), and 4.67 × 10^6^/μL (range, 4.1 × 10^6^/μL to 5.1 × 10^6^/μL), respectively. No activation of the LP-PRP was required with this commercial system. This preparation yielded approximately 5 mL of LP-PRP, which was then injected into the symptomatic knee under ultrasound guidance. Patients were asked to follow a 1-week restricted weight-bearing program with crutches, and then a 1-week period of activities of daily living without resuming a formal exercise program. Afterwards, physical therapy was prescribed to all patients once a week for 8 weeks. Range of motion and isometric strengthening exercises were initiated 2 weeks after the injection. Gradual resistance strengthening was started 4 to 6 weeks after the injection. Eccentric strengthening exercises were restricted for 6 weeks after the injection to avoid excessive loads placed on the joint. Nonsteroidal anti-inflammatory drugs (NSAIDs) and alcohol were restricted 1 week before and 3 days after the injection. No adverse events related to the LP-PRP injection were observed.

### Patient-Reported Outcome Measures

Questionnaires (Knee Osteoarthritis Outcome Score [KOOS], International Knee Documentation Committee [IKDC], and Short Form-12 [SF-12]) were administered to patients before the injection and at 3 months, 6 months, and 12 months after the injection. The KOOS survey assesses 5 categories: Pain, Symptoms, Activities of Daily Living (ADL), Sports and Recreation Function, and Knee-Related Quality of Life (QOL). The scale ranges from 0 to 100, with 0 being the worst and 100 being the best. Similarly, the IKDC assesses domains of pain, symptoms, sports, and function. The scale ranges from 0 to 100, with 0 being the worst and 100 being the best. The SF-12 physical (PCS) and mental component summary (MCS) evaluates the patient’s physical and mental health, respectively. Both PCS and MCS scales are transformed to have a mean of 50 and a standard deviation of 10 in the general US population.

### MRI Acquisition and Assessment

All images were acquired using a 3-T MRI scanner (GE HealthCare, Chicago, IL) with an 8-channel knee coil (Invivo Corporation, Gainesville, FL). High-resolution, 3D fast spin-echo (CUBE) images were acquired to evaluate cartilage, meniscus, and bone morphology. The imaging parameters included repetition time = 1200 milliseconds, echo time = 26 milliseconds, echo train length = 32 milliseconds, field of view = 36 cm, matrix = 512 × 512, slice thickness = 0.6 mm, acquisition time = 8 minutes, 12 seconds. Sagittal T1ρ- and T2-weighted sequence were obtained using a previously developed method based on combined T1ρ and T2 acquisition techniques.^19^ The imaging parameters included: repetition time/echo time = 6.3/12.8 milliseconds, field of view = 36 cm, matrix = 256 × 256, and slice thickness = 4 mm; T1ρ time of spin-lock = 0, 10, 40, 80 milliseconds; T2 preparation TE = 0, 13, 26, 51 milliseconds; and acquisition time = 8 minutes, 28 seconds.

All images were evaluated by an orthopaedic surgeon who was previously trained by musculoskeletal radiologists and research scientists in our biomedical imaging department and specializes in QMRI research (F.S.). A Whole-Organ MRI Scoring (WORMS) system was used to assess cartilage, menisci, and knee effusion at baseline and 6-month follow-up.^20^

### Image Postprocessing

After image acquisition, the CUBE images were registered and down-sampled in the sagittal direction to match the first T1ρ echo. Cartilage was segmented semiautomatically on CUBE into 6 compartments (lateral femur condyle, lateral tibia, medial femur condyle, medial tibia, trochlea, and patella) using an in-house program developed with MATLAB (Mathworks, Natick, MA). Care was taken not to include subchondral plate and synovial fluid in the segmentations. Mean T1ρ and T2 values were calculated for each cartilage compartment after transferring the segmentations from CUBE onto the maps.

### Statistical Analysis

Based on previous QMRI analysis of knee cartilage lesions, an a priori power analysis was performed for a one-tailed analysis with alpha = 0.05 and beta = 0.80 which revealed a required sample size of 10 patients to identify a significant relationship with differences of 6 milliseconds in T1ρ and/or T2 relaxation times.^21^ Minimum clinical important difference (MCID) for the outcome measures at 12 months was calculated using the statistical distribution method and was defined as ½ the standard deviation of change.^22^ T1ρ and T2 values for regions with and without cartilage lesions identified on WORMS was averaged for each subject. Kruskal–Wallis signed rank tests were used to compare changes in T1ρ and T2 values from baseline to 6-month follow-up. Spearman correlations were used to determine the relationship between changes in T1ρ and T2 values and changes in patient-reported outcome measures from baseline to 6-month follow-up and from baseline to 12-month follow-up. All statistical analyses were performed using SPSS Statistics, version 28.0 (IBM Corp., Armonk, NY). For comparisons between change in T1ρ or T2 values over time, the significance level was set at 0.05. To account for multiple comparisons made for correlations between change in T1ρ or T2 values and the 8 different patient-reported outcomes, Bonferroni correction was applied, and the significance level was set to .006.

## Results

### Baseline Clinical and Imaging Characteristics

Ten patients were enrolled (9 female, 1 male) with a mean age of 52.9 years (range, 42-68 years) and mean body mass index of 23.2 ± 1.9. Radiographs showed KL grade 1 in 1 patient and grade 2 in 9 patients. Based on MRI evaluation of the ipsilateral knee, 9 patients had patellar cartilage lesions, whereas 7 patients had trochlear cartilage lesions (Table 1). In the medial compartment, 3 patients had cartilage lesions in the medial femur, whereas only 2 patients had lesions in the medial tibia. On the lateral side, 1 patient had a cartilage lesion of the lateral femur, and 2 patients had cartilage lesion of the lateral tibia (Table 1). Three patients had degenerative meniscal tears in the ipsilateral knee (2 medial meniscus, 1 lateral meniscus). An effusion was present in 7 knees (grade 1: n = 3, grade 2: n = 4).

**Table 1:** WORMS of Cartilage Lesions in Ipsilateral and Contralateral Knee Over Time

	Baseline (n = 10)							6 Months (n = 9)						
	Global	PAT	TrF	MF	MT	LF	LT	Global	PAT	TrF	MF	MT	LF	LT
Ipsilateral	8.8 (range, 3-26)							9.8 (range, 2-26)						
Grade 0		1 (10%)	4 (40%)	7 (70%)	8 (80%)	9 (90%)	8 (80%)		1 (11%)	3 (33%)	6 (67%)	7 (78%)	7 (78%)	3 (33%)
Grade 1		0 (0%)	0 (0%)	0 (0%)	0 (0%)	0 (0%)	0 (0%)		1 (11%)	0 (0%)	0 (0%)	0 (0%)	0 (0%)	1 (11%)
Grade 2		1 (10%)	0 (0%)	0 (0%)	0 (0%)	0 (0%)	1 (10%)		1 (11%)	1 (11%)	1 (11%)	0 (0%)	1 (11%)	3 (33%)
Grade 2.5		2 (20%)	4 (40%)	1 (10%)	0 (0%)	0 (0%)	0 (0%)		2 (22%)	3 (33%)	1 (11%)	0 (0%)	0 (0%)	1 (11%)
Grade 3		1 (10%)	0 (0%)	0 (0%)	0 (0%)	0 (0%)	0 (0%)		0 (0%)	0 (0%)	0 (0%)	0 (0%)	0 (0%)	0 (0%)
Grade 4		1 (10%)	0 (0%)	1 (10%)	1 (10%)	1 (10%)	1 (10%)		1 (11%)	0 (0%)	1 (11%)	1 (11%)	1 (11%)	1 (11%)
Grade 5		4 (40%)	2 (20%)	1 (10%)	1 (10%)	0 (0%)	0 (0%)		4 (44%)	2 (22%)	0 (0%)	1 (11%)	0 (0%)	0 (0%)
Grade 6		0 (0%)	0 (0%)	0 (0%)	0 (0%)	0 (0%)	0 (0%)		0 (0%)	0 (0%)	0 (0%)	0 (0%)	0 (0%)	0 (0%)

	Baseline (n = 10)							6-Months (n = 8)						
	Global	PAT	TrF	MF	MT	LF	LT	Global	PAT	TrF	MF	MT	LF	LT
Contralateral	7.1 (range, 0-25)							8.9 (range, 1-25)						
Grade 0		3 (30%)	5 (50%)	7 (70%)	9 (90%)	9 (90%)	6 (60%)		0 (0%)	3 (38%)	5 (63%)	7 (88%)	7 (88%)	4 (50%)
Grade 1		0 (0%)	0 (0%)	0 (0%)	0 (0%)	0 (0%)	0 (0%)		0 (0%)	1 (12%)	0 (0%)	0 (0%)	0 (0%)	0 (0%)
Grade 2		0 (0%)	2 (20%)	0 (0%)	0 (0%)	0 (0%)	2 (20%)		1 (12%)	1 (12%)	0 (0%)	0 (0%)	0 (0%)	2 (25%)
Grade 2.5		2 (20%)	1 (10%)	2 (20%)	0 (0%)	0 (0%)	1 (10%)		4 (50%)	1 (12%)	2 (25%)	0 (0%)	0 (0%)	1 (12%)
Grade 3		1 (10%)	0 (0%)	0 (0%)	1 (10%)	1 (10%)	0 (0%)		0 (0%)	0 (12%)	0 (0%)	1 (12%)	1 (12%)	0 (0%)
Grade 4		0 (0%)	2 (20%)	1 (10%)	0 (0%)	0 (0%)	0 (0%)		0 (0%)	0 (0%)	1 (12%)	0 (0%)	0 (0%)	0 (0%)
Grade 5		4 (40%)	0 (0%)	0 (0%)	0 (0%)	0 (0%)	1 (10%)		3 (38%)	2 (25%)	0 (0%)	0 (0%)	0 (0%)	1 (12%)
Grade 6		0 (0%)	0 (0%)	0 (0%)	0 (0%)	0 (0%)	0 (0%)		0 (0%)	0 (0%)	0 (0%)	0 (0%)	0 (0%)	0 (0%)

LF, lateral femur; LT, lateral tibia; MF, medial femur; MRI, magnetic resonance imaging; MT, medial tibia; PAT, patella; TrF, trochlea; WORMS, Whole-Organ Magnetic Resonance Imaging Score.

### Patient-Reported Outcome Scores

**Fig 1:**
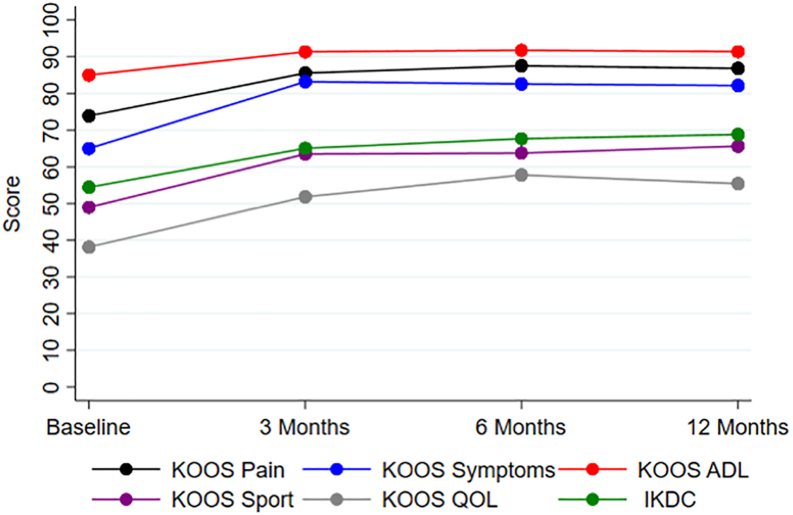
Change in Knee-injury Osteoarthritis Outcome Score (KOOS) and International Knee Documentation Committee (IKDC) scores over time.

The baseline and follow-up outcome scores for KOOS and IKDC are shown in Fig 1 and Table 2. From baseline to 3 months after injection, KOOS in all subscales and IKDC significantly improved (all *P* < .05). This improvement was sustained in all measures at 6 months and 12 months except for the KOOS sports subscale (*P* = .065 and .051, respectively). There was no significant change in SF-12 PCS or MCS from baseline to 12-month follow-up (PCS: 46.7 ± 9.7 vs 53.5 ± 4.7, *P* = .161; MCS: 51.1 ± 8.3 vs 49.5 ± 6.0, *P* = .484). The MCID value at 12 months was 4.3 points for KOOS pain, 4.5 points for KOOS symptoms, 2.9 points for KOOS ADL, 6.9 points for KOOS sports, 9.2 points for KOOS QOL, 3.1 points for IKDC, 4.3 points for SF-12 PCS, and 4.3 points for SF-12 MCS. MCID was achieved for 75% of patients for KOOS pain, 87.5% of patients for KOOS symptoms, 87.5% of patients for KOOS ADL, 62.5% of patients for KOOS sports, 62.5% of patients for KOOS QOL, 87.5% of patients for IKDC, 37.5% of patients for SF-12 PCS, and 25% of patients for SF-12 MCS.

**Table 2:** Change in Patient-Reported Outcomes Over Time

	KOOS					IKDC	SF-12	
	Pain	Symptoms	ADL	Sport	QOL		PCS	MCS
Baseline	73.9 ± 10.4	65.0 ± 8.9	85.0 ± 8.8	49.0 ± 19.7	38.1 ± 16.5	54.5 ± 12.1	46.7 ± 9.7	51.1 ± 8.3
3 mo	85.6 ± 8.8	83.2 ± 6.9	91.3 ± 6.8	63.5 ± 20.6	51.9 ± 19.1	65.1 ± 11.2	50.6 ± 8.2	51.6 ± 6.6
6 mo	87.5 ± 8.9	82.6 ± 7.3	91.7 ± 6.1	63.8 ± 16.9	57.8 ± 18.8	67.7 ± 11.0	50.0 ± 10.9	53.1 ± 4.8
12 mo	86.8 ± 11.2	82.1 ± 9.9	91.4 ± 7.3	65.6 ± 20.4	55.5 ± 18.4	68.8 ± 7.7	53.5 ± 4.7	49.5 ± 6.0
*P* value∗	**.036**	**.018**	**.025**	.051	**.011**	**.017**	.161	.484

NOTE. Values in bold denotes significance.

ADL, activities of daily living; IKDC, International Knee Documentation Committee; KOOS, Knee Osteoarthritis Outcome Score; MCS, mental component score; PCS, physical component score; QOL, quality of life; SF-12, Short Form-12.

∗ Comparison between baseline and 12-month scores.

### WORMS and Cartilage T1ρ and T2 after PRP Injection

At baseline, there was no significant difference in WORMS cartilage lesion score between the ipsilateral and contralateral knee (8.8 [range, 3-26] vs 7.1 [range, 0-25], *P* = .185). T1ρ and T2 values in the affected compartments of the ipsilateral knee were significantly greater than that of the unaffected compartments (T1ρ: 46.5 ± 3.5 milliseconds vs 43.0 ± 2.3 milliseconds, *P* = .036; T2: 33.6 ± 3.4 milliseconds vs 32.2 ± 4.0 milliseconds, *P* = .028) (Table 3). The T1ρ and T2 values of the affected compartments of the ipsilateral knee were not significantly different from that of the contralateral knee (T1ρ: *P* = .401; T2: *P* = .484).

**Table 3:** T1ρ and T2 Values in Ipsilateral and Contralateral Knees

		Baseline	T1ρ, milliseconds	*P* Value	Baseline	T2ρ, milliseconds	*P* Value
			6 Months			6 Months	
Ipsilateral	Affected compartments	46.5 ± 3.5	43.7 ± 3.5	**.036**	33.6 ± 3.4	31.2 ± 3.6	**.017**
	Unaffected compartments	43.0 ± 2.3	43.8 ± 3.5	.612	31.7 ± 2.5	31.4 ± 2.5	.499
Contralateral	Affected compartments	45.0 ± 2.2	44.0 ± 2.9	.401	32.2 ± 4.0	31.8 ± 3.1	.779
	Unaffected compartments	43.6 ± 3.1	43.2 ± 3.3	.612	32.7 ± 2.7	32.0 ± 3.2	.398

NOTE. Values in bold denotes significance.

**Fig 2:**
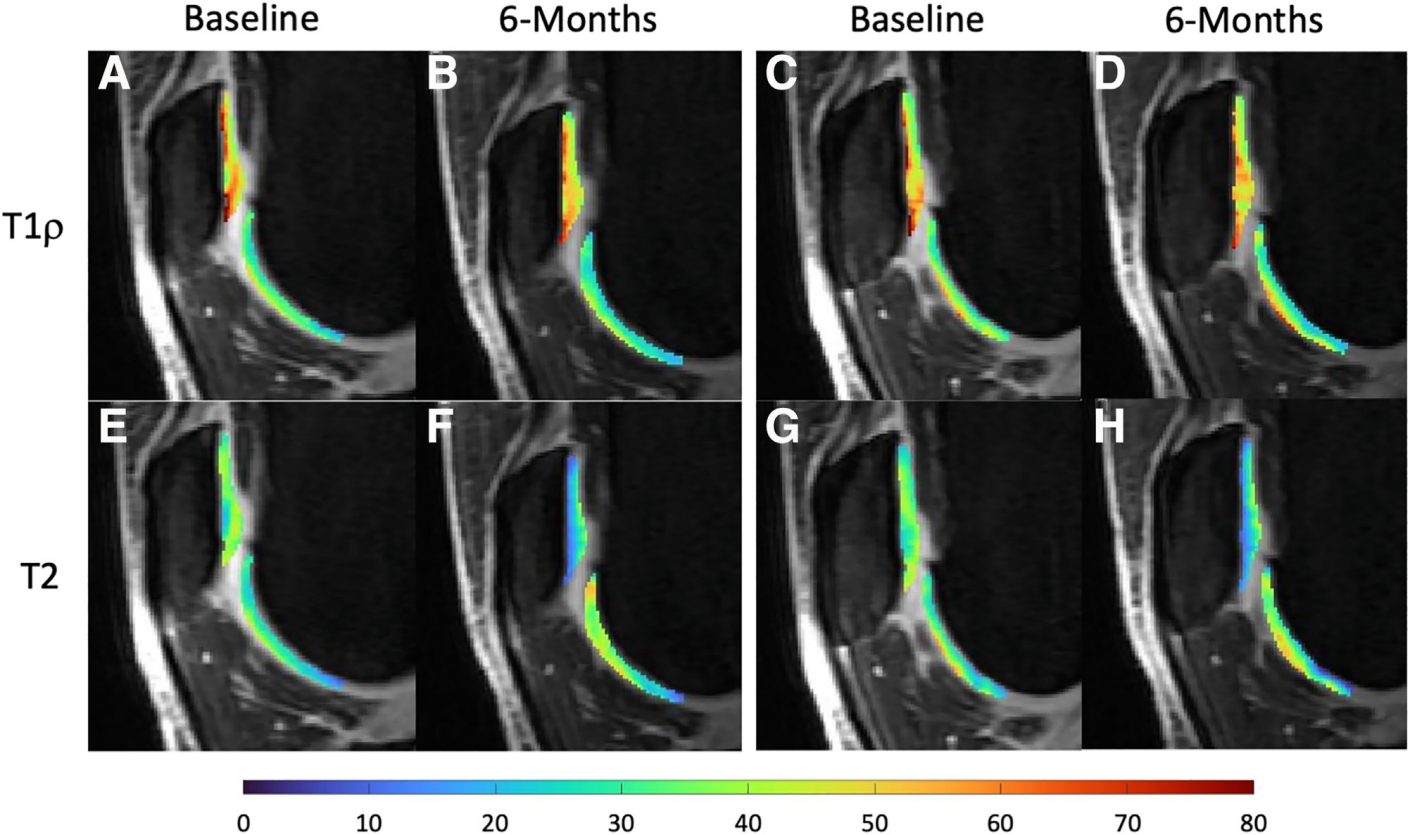
Sagittal (A-D) T1ρ and (E-H) T2 maps of the patellofemoral compartment in the symptomatic knee at baseline and 6 months after injection for 2 corresponding and consecutive slices. The T1ρ and T2 values in the patella of this patient decreased by 8.2 milliseconds and 5.5 milliseconds, respectively.

At 6-month follow-up, there was no significant change in the WORMS cartilage lesion scores in either knee compared with baseline (ipsilateral: 8.8 [range, 3-26] vs 9.8 [range, 2-26], *P* = .671; contralateral: 7.1 [range, 0-25] vs 8.9 [range, 1-25], *P* = .180). T1ρ values significantly decreased in the affected compartments of the ipsilateral knee from baseline to 6 months (46.5 ± 3.5 milliseconds vs 43.7 ± 3.5 milliseconds, *P* = .036) (Fig 2). Similarly, T2 values at 6 months were also significantly lower in the affected compartments of the ipsilateral knee compared with baseline (33.6 ± 3.4 milliseconds vs 31.2 ± 3.6 milliseconds, *P* = .017). There was no significant change in the T1ρ or T2 values from baseline to 6 months in the unaffected compartments of the ipsilateral knee or in the affected and unaffected compartments of the contralateral knee. There was no significant correlation between the 6-month change in T1ρ or T2 values of the affected compartments and the 6-month or 12-month change in patient-reported outcomes (all *P* > .006) (Table 4).

**Table 4:** Correlations Between 6-Month Change in T1ρ or T2 and 6-Month and 12-Month Change in Patient-Reported Outcomes

	KOOS					Δ IKDC	SF-12	
	Δ Pain	Δ Symptoms	Δ ADL	Δ Sports	Δ QOL		Δ PCS	Δ MCS
6 mo								
Δ T1ρ affected	r = –0.250 *P* = .589	r = –0.630 *P* = .129	r = –0.143 *P* = .760	r = –0.236 *P* = .610	r = 0.037 *P* = .937	r = –0.179 *P* = .702	r = 0.143 *P* = .760	r = 0.143 *P* = .760
Δ T2 affected	r = –0.286 *P* = .535	r = –0.482 *P* = .274	r = 0.464 *P* = .294	r = –0.164 *P* = .726	r = 0.094 *P* = .842	r = –0.571 *P* = .180	r = 0.643 *P* = .119	r = –0.464 *P* = .294
12 mo								
Δ T1ρ affected	r = –0.464 *P* = .294	r = –0.286 *P* = .535	r = –0.357 *P* = .432	r = 0.018 *P* = .969	r = –0.312 *P* = .496	r = 0.179 *P* = .702	r = –0.143 *P* = .760	r = 0.821 *P* = .023
Δ T2 affected	r = –0.036 *P* = .939	r = –0.750 *P* = .052	r = 0.500 *P* = .253	r = 0.018 *P* = .969	r = –0.330 *P* = .469	r = –0.500 *P* = .253	r = –0.036 *P* = .939	r = –0.357 *P* = .432

NOTE. Significance was set at a *P* value of .006.

ADL, activities of daily living; IKDC, International Knee Documentation Committee; KOOS, Knee Osteoarthritis Outcome Score; MCS, mental component score; PCS, physical component score; QOL, quality of life; SF-12, Short Form-12.

## Discussion

In this pilot study of patients with early knee OA, both T1ρ and T2 values decreased in the affected cartilage compartments 6 months after LP-PRP injection. This may reflect a possible beneficial effect of LP-PRP therapy on altering the course of cartilage degeneration. Furthermore, patient-reported outcomes demonstrated significant improvements and were sustained 12 months after injection. These improvements in patient-reported outcomes, however, were not correlated with the changes in the cartilage biochemical matrix as assessed by T1ρ and T2 MRI.

There are few studies that have evaluated the effect of PRP injections on cartilage using QMRI. In an anterior cruciate transection rodent model designed to represent early OA progression, Huang et al.^23^ found that T2∗ decreased at 10 weeks in the PRP group compared with placebo, suggesting that PRP has a chondroprotective and collagen-loss preventive effect in cartilage. Bellisari et al.^15^ used T2 MRI to evaluate the effect of 3 PRP injections on patellofemoral cartilage lesions in 34 patients at a mean follow-up of 6.4 months. The mean T2 relaxation time of the global patellofemoral joint improved by 6.0%.^15^ These studies are in accordance with our results, which also showed a significant T1ρ decrease of 6.0% and T2 decrease of 7.1% in the affected compartments after LP-PRP injection. Interestingly, our study also adds that both the T1ρ and T2 values of the cartilage of unaffected compartments had no change after injection, which may suggest that the increased proteoglycan and collagen synthesis is limited to the regions of cartilage injury.

In contrast to the aforementioned findings, several studies found no difference in T2 values of knee chondral lesions after treatment with a combination of PRP and mesenchymal stem cells or microfat.^16^^,^^17^ Pintat et al.^17^ performed a single injection of mesenchymal stem cells and LP-PRP in 19 patients with patellofemoral chondral lesions and found no significant change in T2 values at 6 months. In their study, the mean T2 value of the global patellofemoral joint was 54.7 milliseconds, which is much greater than the mean T2 value of 31.2 milliseconds reported in the current study.^17^ This large T2 value may indicate that the patellofemoral cartilage is so severely degenerated that it lacks the potential for healing. Our study limited participants to those with early knee OA (KL grade 1 or 2), which may have accounted for the greater likelihood of cartilage modification in relaxation times compared with studies that included severe degeneration. In a double-blind randomized comparative trial, 30 patients with knee OA were randomized to receive a single microfat injection or a combined injection of LP-PRP and microfat.^16^ At 6-month follow-up, maximum T2 values decreased compared with baseline in the microfat only group, but not in the LP-PRP and microfat groups.^16^ Aside from the differences in injection, the discrepancy between these findings and the current study could be due to differences in image postprocessing. Only a single 3-mm slice focused on the chondral defect was segmented in their study, which may not be representative of the entire chondral lesion.

Although improvement in the cartilage biochemical matrix was observed using T1ρ and T2 mapping, there was no change in the morphologic grading of the cartilage lesions in the symptomatic ipsilateral knee. Furthermore, there was also no difference in the WORMS cartilage score between the ipsilateral and contralateral knee at any time point. These findings suggest that LP-PRP injections do not result in any cartilage thickening of the chondral lesions and are consistent with the results of several studies.^17^^,^^24^, ^25^, ^26^, ^27^, ^28^ In a recent randomized control trial comparing 3 injections of LP-PRP to saline in patients with mild-to-moderate medial knee OA, there was no significant difference in the change in medial tibial cartilage volume in both groups at 12 months.^26^ Similarly, Buendía-López et al.^25^ found no change in tibiofemoral cartilage thickness on MRI at 12 months in patients who were randomized to LP-PRP, hyaluronic acid, or NSAIDs. Conversely, other studies have reported increased knee cartilage volume and thickness after LP-PRP injection.^15^^,^^16^^,^^29^^,^^30^ The differences in outcomes among studies may be due to heterogeneity in MRI techniques, PRP preparation, number of injections, follow-up time, and severity of chondral lesions. There is currently insufficient evidence to support that PRP therapy can regenerate and regrow new cartilage in patients.

There was significant improvement in patient-reported outcomes 3 months after LP-PRP injection and the improvement was sustained at 12 months. Moreover, MCID was achieved in more than one half of patients in KOOS subscales and IKDC. These findings are corroborated by meta-analyses of 6 randomized controlled trials, which reported a weighted mean improvement of 38% in IKDC scores.^9^ In a cohort of 215 patients receiving LP-PRP injection, Boffa et al.^31^ similarly found that the likelihood of achieving MCID in KOOS or IKDC was 89.8% at 6 months and 85.6% at 12 months. Interestingly, no significant correlations were identified between the change in patient-reported outcomes and the change in T1ρ or T2 imaging biomarkers after LP-PRP injection. The lack of a significant association may be due to the small sample size as several previous studies identified correlations between T1ρ measurements of chondral lesions and the severity of patient symptoms and function.^13^^,^^32^ Alternatively, the favorable effect of PRP therapy on patient-reported outcomes may also be due to the anti-inflammatory effects rather than increased proteoglycan and collagen deposition in the areas of cartilage damage.

### Limitations

Although this study demonstrated that LP-PRP injections improved the cartilage biochemical composition in patients with early knee OA, it is not without limitations. First, the study had a small sample size of 8 patients with MRI follow-up at baseline and 6 months. Post-hoc power analysis revealed that a sample size of 45 patients would be needed to detect a significant correlation between the change T1ρ or T2 imaging biomarkers and patient-reported outcomes. It is also unknown whether the alterations in the cartilage matrix are transitory or persist at longer follow-ups. Second, most of the patients in this study were middle-aged women with KL grade 1-2 patellofemoral disease. Thus, these results should not be extrapolated to male patients with chondral lesions in other compartments, patients with more severe OA, or older patients. Furthermore, subcompartment, laminar analysis, or voxel-based relaxometry of the cartilage was not performed due to risk of multiple comparisons with our limited sample size. As such, our T1ρ and T2 measurements may have lacked the sensitivity of detecting even more subtle changes. Another limitation was that the LP-PRP composition was not reported using a standard biologics classification system.^33^ Furthermore, NSAIDs were only restricted for 3 days after injection and antiplatelet medication use was not recorded. Changes in patient-reported outcomes and cartilage T1ρ and T2 may be potentially influenced by the early resumption of NSAIDs. In addition, it was unknown whether patients were taking any analgesic medications at various time points, which may have confounded our results. Finally, one variable that is difficult to standardize is patient activity level after the completion of physical therapy and the effects of increased or decreased exercise. Previous studies have demonstrated alterations in relaxation times due to level of physical activity.^34^^,^^35^ However, using the contralateral knee as a control for comparison in this study helped to decrease confounding from this variable.

## Conclusions

Patients undergoing LP-PRP injections for the treatment of mild-to-moderate knee OA had increased proteoglycan and collagen deposition in the cartilage of affected compartments by 6 months after injection. Patient-reported outcomes scores improved 3 months after injection and were sustained through 1 year after injection, but these improvements were not associated with the changes in proteoglycan and collagen deposition in knee cartilage.

## Supplementary Data

ICMJE author disclosure forms

## References

[1] Murray C.J., Atkinson C., Bhalla K. (2013). The state of US health, 1990-2010: Burden of diseases, injuries, and risk factors. JAMA.

[2] Lawrence R.C., Felson D.T., Helmick C.G. (2008). Estimates of the prevalence of arthritis and other rheumatic conditions in the United States. Part II. Arthritis Rheum.

[3] Felson D.T., Lawrence R.C., Hochberg M.C. (2000). Osteoarthritis: New insights. Part 2: treatment approaches. Ann Intern Med.

[4] Wallace I.J., Worthington S., Felson D.T. (2017). Knee osteoarthritis has doubled in prevalence since the mid-20th century. Proc Natl Acad Sci U S A.

[5] Sundman E.A., Cole B.J., Fortier L.A. (2011). Growth factor and catabolic cytokine concentrations are influenced by the cellular composition of platelet-rich plasma. Am J Sports Med.

[6] Dragoo J.L., Braun H.J., Durham J.L. (2012). Comparison of the acute inflammatory response of two commercial platelet-rich plasma systems in healthy rabbit tendons. Am J Sports Med.

[7] Mazzocca A.D., McCarthy M.B., Chowaniec D.M. (2012). Platelet-rich plasma differs according to preparation method and human variability. J Bone Joint Surg Am.

[8] Dai W.L., Zhou A.G., Zhang H., Zhang J. (2017). Efficacy of platelet-rich plasma in the treatment of knee osteoarthritis: A meta-analysis of randomized controlled trials. Arthroscopy.

[9] Belk J.W., Kraeutler M.J., Houck D.A., Goodrich J.A., Dragoo J.L., McCarty E.C. (2021). Platelet-rich plasma versus hyaluronic acid for knee osteoarthritis: A systematic review and meta-analysis of randomized controlled trials. Am J Sports Med.

[10] Eckstein F., Burstein D., Link T.M. (2006). Quantitative MRI of cartilage and bone: Degenerative changes in osteoarthritis. NMR Biomed.

[11] Andreisek G., White L.M., Sussman M.S. (2009). Quantitative MR imaging evaluation of the cartilage thickness and subchondral bone area in patients with ACL-reconstructions 7 years after surgery. Osteoarthritis Cartilage.

[12] Su F., Hilton J.F., Nardo L. (2013). Cartilage morphology and T1rho and T2 quantification in ACL-reconstructed knees: A 2-year follow-up. Osteoarthritis Cartilage.

[13] Su F., Pedoia V., Teng H.L. (2016). The association between MR T1rho and T2 of cartilage and patient-reported outcomes after ACL injury and reconstruction. Osteoarthritis Cartilage.

[14] Duvvuri U., Reddy R., Patel S.D., Kaufman J.H., Kneeland J.B., Leigh J.S. (1997). T1rho-relaxation in articular cartilage: Effects of enzymatic degradation. Magn Reson Med.

[15] Bellisari F.C., De Marino L., Arrigoni F. (2021). T2-mapping MRI evaluation of patellofemoral cartilage in patients submitted to intra-articular platelet-rich plasma (PRP) injections. Radiol Med.

[16] Louis M.L., Dumonceau R.G., Jouve E. (2021). Intra-articular injection of autologous microfat and platelet-rich plasma in the treatment of knee osteoarthritis: A double-blind randomized comparative study. Arthroscopy.

[17] Pintat J., Silvestre A., Magalon G. (2017). Intra-articular injection of mesenchymal stem cells and platelet-rich plasma to treat patellofemoral osteoarthritis: Preliminary results of a long-term pilot study. J Vasc Interv Radiol.

[18] Turzi A., Toit D.F.D. (2013). Cell preparations for extemporaneous use, useful for healing and rejuvenation in vivo. US Patent and Tracking Office.

[19] Li X., Wyatt C., Rivoire J. (2014). Simultaneous acquisition of T1rho and T2 quantification in knee cartilage: Repeatability and diurnal variation. J Magn Reson Imaging.

[20] Peterfy C.G., Guermazi A., Zaim S. (2004). Whole-Organ Magnetic Resonance Imaging Score (WORMS) of the knee in osteoarthritis. Osteoarthritis Cartilage.

[21] Theologis A.A., Haughom B., Liang F. (2014). Comparison of T1rho relaxation times between ACL-reconstructed knees and contralateral uninjured knees. Knee Surg Sports Traumatol Arthrosc.

[22] Copay A.G., Chung A.S., Eyberg B., Olmscheid N., Chutkan N., Spangehl M.J. (2018). Minimum clinically important difference: Current trends in the orthopaedic literature, part I: Upper extremity: A systematic review. JBJS Rev.

[23] Huang G.S., Peng Y.J., Hwang D.W. (2021). Assessment of the efficacy of intra-articular platelet rich plasma treatment in an ACLT experimental model by dynamic contrast enhancement MRI of knee subchondral bone marrow and MRI T2 (∗) measurement of articular cartilage. Osteoarthritis Cartilage.

[24] Halpern B., Chaudhury S., Rodeo S.A. (2013). Clinical and MRI outcomes after platelet-rich plasma treatment for knee osteoarthritis. Clin J Sport Med.

[25] Buendia-Lopez D., Medina-Quiros M., Fernandez-Villacanas Marin M.A. (2018). Clinical and radiographic comparison of a single LP-PRP injection, a single hyaluronic acid injection and daily NSAID administration with a 52-week follow-up: A randomized controlled trial. J Orthop Traumatol.

[26] Bennell K.L., Paterson K.L., Metcalf B.R. (2021). Effect of intra-articular platelet-rich plasma vs placebo injection on pain and medial tibial cartilage volume in patients with knee osteoarthritis: The RESTORE randomized clinical trial. JAMA.

[27] Hart R., Safi A., Komzak M., Jajtner P., Puskeiler M., Hartova P. (2013). Platelet-rich plasma in patients with tibiofemoral cartilage degeneration. Arch Orthop Trauma Surg.

[28] Moretti L., Maccagnano G., Coviello M. (2022). Platelet rich plasma injections for knee osteoarthritis treatment: A prospective clinical study. J Clin Med.

[29] Raeissadat S.A., Ghorbani E., Sanei Taheri M. (2020). MRI changes after platelet rich plasma injection in knee osteoarthritis (randomized clinical trial). J Pain Res.

[30] Samara O., Al-Ajlouni J., Al-Najar M. (2017). Intra-articular autologous platelet lysates produce positivee MRI structural changes in early and inter-mediate knee osteoarthrosis. Pakistan J Radiol.

[31] Boffa A., Andriolo L., Franceschini M. (2021). Minimal clinically important difference and patient acceptable symptom state in patients with knee osteoarthritis treated with PRP injection. Orthop J Sports Med.

[32] Souza R.B., Feeley B.T., Zarins Z.A., Link T.M., Li X., Majumdar S. (2013). T1rho MRI relaxation in knee OA subjects with varying sizes of cartilage lesions. Knee.

[33] Mautner K., Malanga G.A., Smith J. (2015). A call for a standard classification system for future biologic research: The rationale for new PRP nomenclature. PM R.

[34] Kumar D., Souza R.B., Singh J. (2014). Physical activity and spatial differences in medial knee T1rho and t2 relaxation times in knee osteoarthritis. J Orthop Sports Phys Ther.

[35] Lin W., Alizai H., Joseph G.B. (2013). Physical activity in relation to knee cartilage T2 progression measured with 3 T MRI over a period of 4 years: Data from the Osteoarthritis Initiative. Osteoarthritis Cartilage.

